# Supplementary material for: The Impact of SRT2104 on Skeletal Muscle Mitochondrial Function, Redox Biology, and Loss of Muscle Mass in Hindlimb Unloaded Rats
Source: Int J Mol Sci. 2023 Jul 6;24(13):11135. doi: 10.3390/ijms241311135 (PMC10342025; doi:10.3390/ijms241311135)
Supplement: Supplementary file 1 [file ijms-24-11135-s001.zip › ijms-2441996-supplementary.pdf]

SUPPLEMENTAL FIGURES

The Impact of SRT2104 on Skeletal Muscle Mitochondrial Function, Redox Biology, and Loss of Muscle Mass in Hindlimb Unloaded Rats

Lauren T. Wesolowski [1], Jessica L. Simons [1], Pier L. Semanchik [1], Mariam A. Othman [2], Joo-Hyun Kim [2], John M. Lawler [2,3], Khaled Y. Kamal [2,*] and Sarah H. White-Springer [1,*]

[1] Department of Animal Science, College of Agriculture and Life Science, Texas A&M University and Texas A&M AgriLife Research, College Station, TX 77843, USA; wesolowski@tamu.edu (L.T.W.); jls97@tamu.edu (J.L.S.); piersemanchik@tamu.edu (P.L.S.)

[2] Department of Kinesiology & Sport Management, School of Education and Human Development, Texas A&M University, College Station, TX 77843, USA; mariam.othman@tamu.edu (M.A.O.); dhwngusdh@tamu.edu (J.H.K.); jml2621@tamu.edu (J.M.L.)

[3] Department of Nutrition, Texas A&M University, College Station, TX 77843, USA

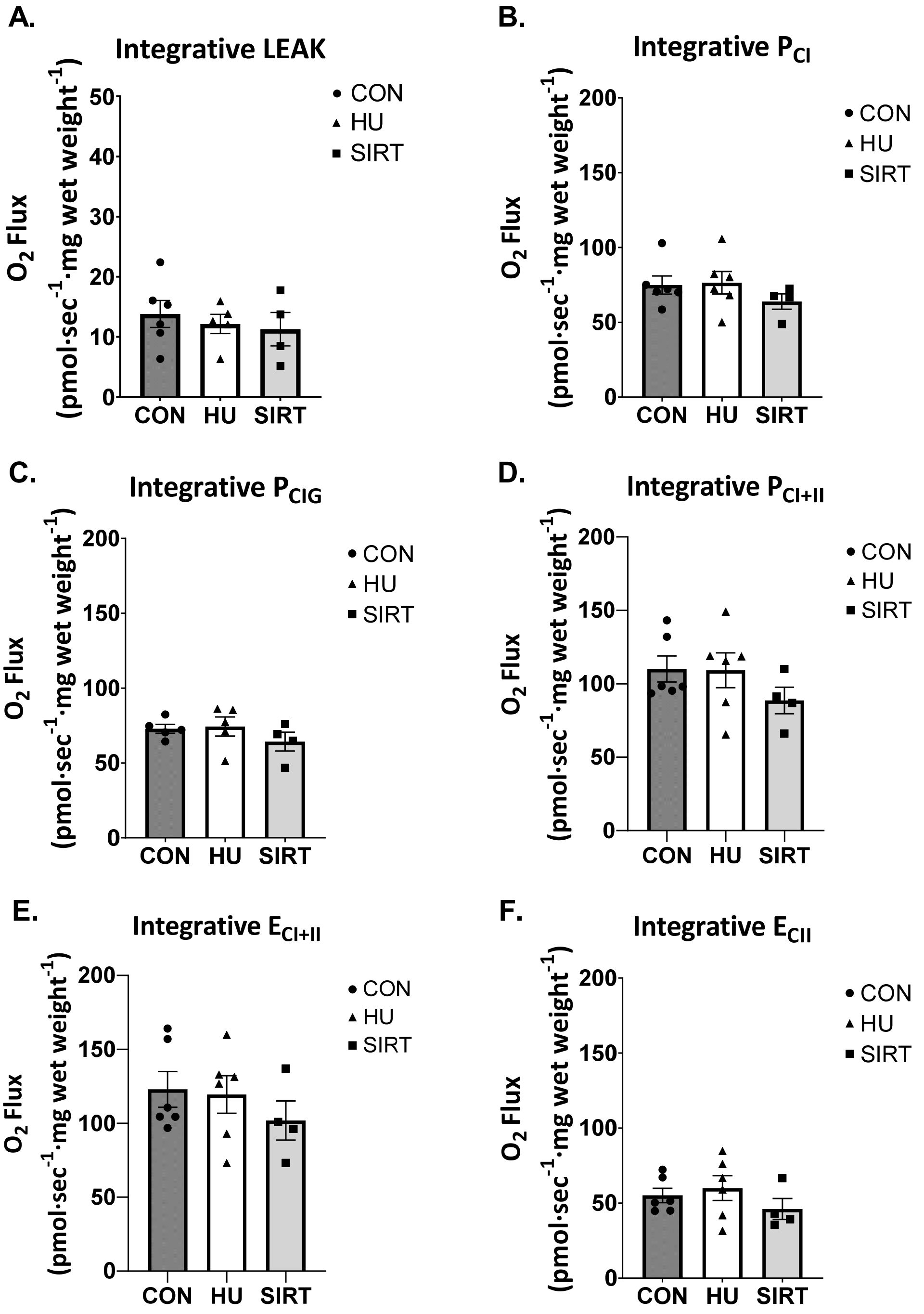

Supplementary Figure S1. Effect of the Sirtuin-1 Agonist SRT2104 on integrative (relative to tissue wet weight) mitochondrial capacities as measured by high-resolution respirometry in the soleus muscle of hindlimb unloaded rats. (A) Integrative proton LEAK, (B, C) oxidative phosphorylation supported by complex I (P_{CI} and P_{CIG}), (D) maximal coupled oxidative phosphorylation (P_{CI+II}), (E) maximal noncoupled electron transfer (E_{CI+II}), and (F) electron transfer supported by complex II only (E_{CII}) capacities in the gastrocnemius muscle of ambulatory control rats (CON; $n = 6$), rats hindlimb unloaded for 10 days (HU; $n = 6$), and rats hindlimb unloaded for 10 days while treated with SRT2104 (SIRT; $n = 4$). Values are means ± SEM.

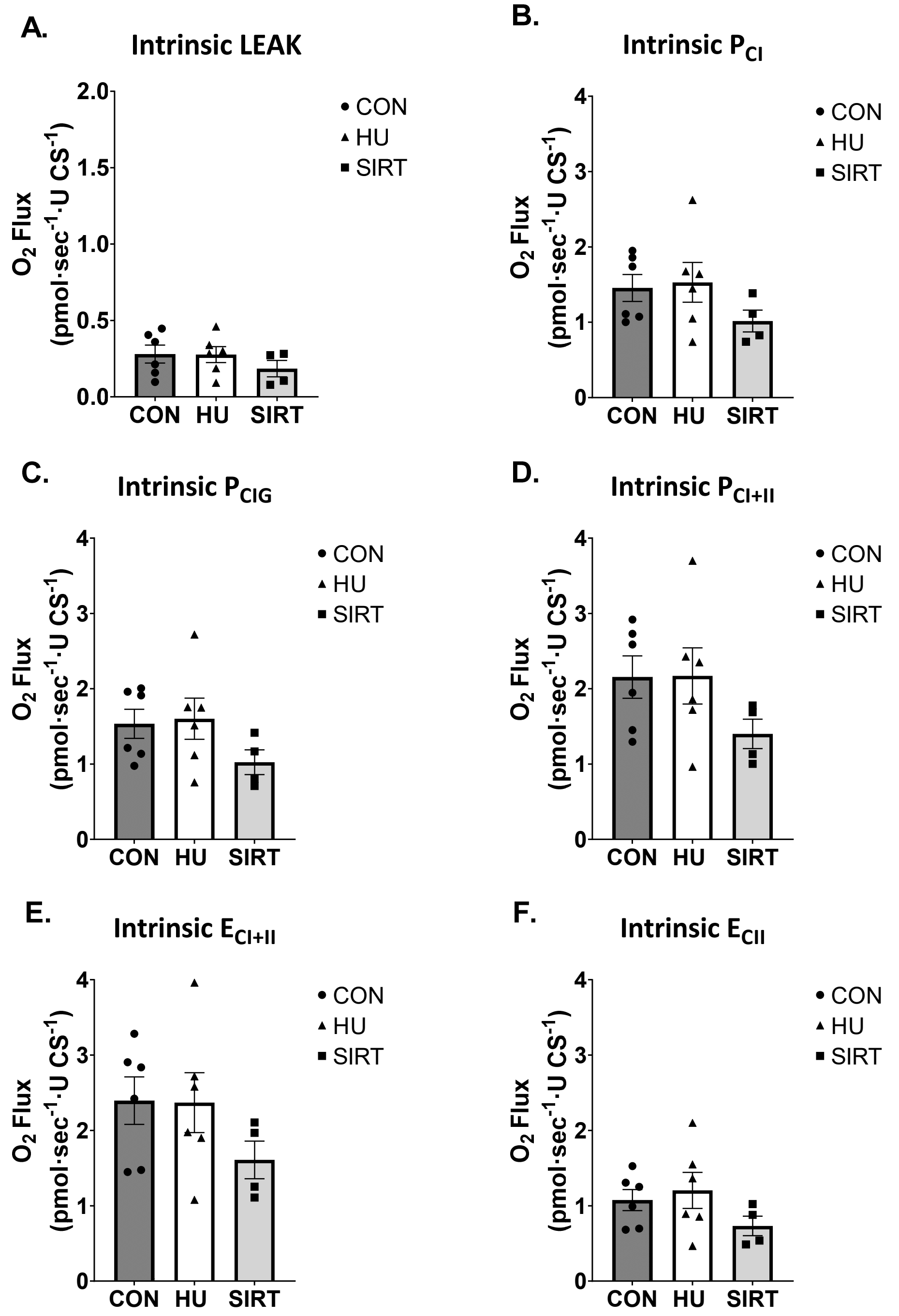

Supplementary Figure S2. Effect of the Sirtuin-1 Agonist SRT2104 on intrinsic (relative to CS activity) mitochondrial capacities as measured by high-resolution respirometry in the soleus muscle of hindlimb unloaded rats. (A) Intrinsic proton LEAK, (B, C) oxidative phosphorylation supported by complex I (P_{CI} and P_{CIG}), (D) maximal coupled oxidative phosphorylation (P_{CI+II}), (E) maximal noncoupled electron transfer (E_{CI+II}), and (F) electron transfer supported by complex II only (E_{CII}) capacities in the gastrocnemius muscle of ambulatory control rats (CON; *n* = 6), rats hindlimb unloaded for 10 days (HU; *n* = 6), and hindlimb unloaded for 10 days while treated with SRT2104 (SIRT; *n* = 4). Values are means ± SEM.

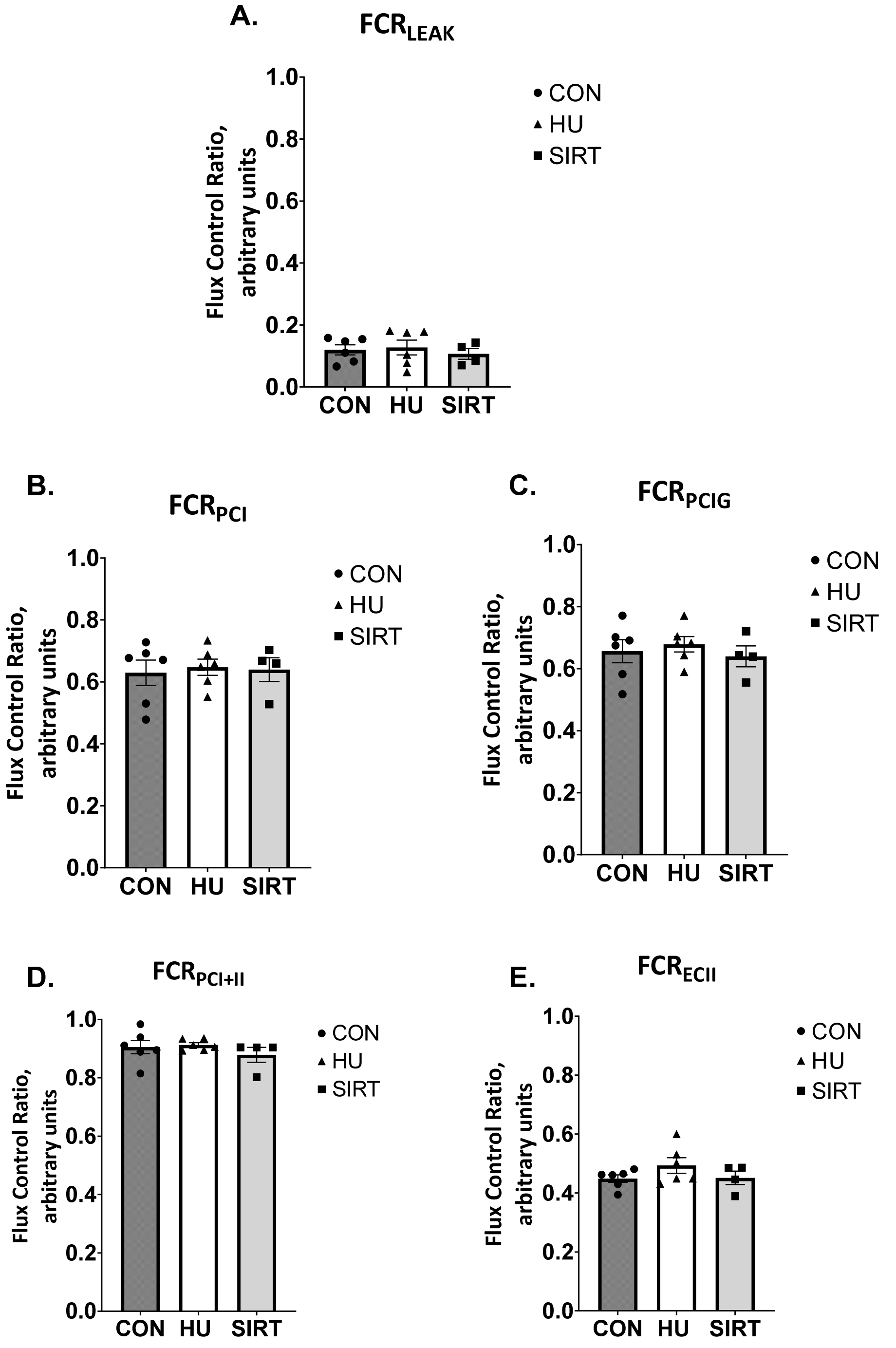

Supplementary Figure S3. Effect of the Sirtuin-1 Agonist SRT2104 on mitochondrial flux control ratios in the soleus muscle of hindlimb unloaded rats. (A) The ratio of LEAK to maximal electron transfer (flux control ratio; FCR_{LEAK}), (B) FCR for oxidative phosphorylation supported by complex I (FCR_{PCI}), (C) FCR when glutamate was added as an additional complex I substrate (FCR_{PCIG}), (D) FCR for maximal coupled oxidative phosphorylation (FCR_{PCI+II}) and (E) FCR for noncoupled electron transfer supported by complex II only (FCR_{ECII}) of ambulatory control rats (CON; n = 6), rats hindlimb unloaded for 10 days (HU; n = 6), and rats hindlimb unloaded for 10 days while treated with SRT2104 (SIRT; n = 4). Values are means ± SEM.
